# Optimal assessment of right ventricular function using cardiac magnetic resonance cine imaging after Mustard palliation for transposition of the great arteries

**DOI:** 10.1186/1532-429X-13-S1-O20

**Published:** 2011-02-02

**Authors:** Laura Jimenez-Juan, Subodh B Joshi, Andrew T Yan, Susan H James, Djeven Deva, Elsie T Nguyen, Sebastian Ley, Andrew M Crean, Narinder Paul, Bernd J Wintersperger, Rachel M Wald

**Affiliations:** 1 Toronto General Hospital, Toronto, ON, Canada; 2 St Michael's Hospital, Toronto, ON, Canada

## Objective

To determine the reproducibility of axial versus short axis methods for measurement of right ventricular volumes and ejection fraction (RVEF) in adults after Mustard palliation for complete transposition of the great arteries (TGA).

## Background

The most common cause of a systemic right ventricle is atrial redirection surgery (Mustard repair) in the setting of complete TGA. Decreased RVEF is common and is a predictor of morbidity and mortality. Cardiac magnetic resonance (CMR) cine imaging is the reference standard for assessment of right heart size and function. However, the optimal method of RV planimetry using CMR in patients with Mustard palliation for complete TGA remains unclear.

## Methods

Consecutive adult patients with atrial redirection surgery (Mustard procedure) for complete TGA were identified from an existing database and steady state free-precession cine images acquired on 1.5 T commercially available scanners were reviewed. To obtain RVEF, both end-diastolic volumes (EDV) and end-systolic volumes (ESV) were measured in two orientations, axial and short axis, by two experienced, independent readers who were blinded to other results. One reader reanalyzed the RV volumes and function ≥ 14 days after the first set of measurements. Intra- and inter-observer variabilities were determined using Bland-Altman analysis.

## Results

A total of 25 patients (16 female, 9 male) were identified (median 31 years, interquartile range 26-37) between 2008 and 2010. All images had sufficient quality for analysis. From the axial stack, the medians (interquartile ranges) were RVEDV 243cc (198-297), RVESV 142cc (101-174) and RVEF 44% (38-50). From the short axis stack, medians (interquartile ranges) were RVEDV 236cc (181-281), RVESV 127cc (90-157) and RVEF 44% (41-50). Volumes derived from short axis and axial stacks were significantly different (p=0.001 for RVEDV and p<0.001 for RVESV) but there was no statistical difference in RVEF between the methods (p=0.38). Bland Altman analysis for evaluation of intra and inter-observer variabilities are summarized in table 1.

**Table 1:** Comparison of axial and short axis RVEF measurements using Bland-Altman analysis

	Axial		Short-axis	
	Intra-observer	Inter-observer	Intra-observer	Inter-observer

Correlation co-efficient (p value)	0.88 (p<0.001)	0.76 (p<0.001)	0.82 (p<0.001)	0.72 (p<0.001)
Bland-Altman bias	0.92	3.29	-0.24	2.23
Limits of agreement	-6.7 , 8.6	-13.9 , 7.4	-12.8 , 12.3	-14.5 - 10.0

## Conclusion

Following Mustard palliation for complete TGA, RVEF measurements derived from axial and short axis stacks are similar, although both RVEDV and RVESV values are significantly higher based on the axial acquisition. Intra-observer measurements appear to be more reliable using the axial as compared with the short axis method.

